# The COVID-19 outbreak and psychiatric hospitals in China: managing challenges through mental health service reform

**DOI:** 10.7150/ijbs.45072

**Published:** 2020-03-15

**Authors:** Yu-Tao Xiang, Yan-Jie Zhao, Zi-Han Liu, Xiao-Hong Li, Na Zhao, Teris Cheung, Chee H. Ng

**Affiliations:** 1 Unit of Psychiatry, Institute of Translational Medicine, Faculty of Health Sciences, University of Macau, Macao SAR, China.; 2 Center for Cognition and Brain Sciences, University of Macau, Macao SAR, China.; 3 Center for Cognition and Brain Disorders, the Affiliated Hospital, Institutes of Psychological Sciences, Hangzhou Normal University, Hangzhou, China.; 4 The National Clinical Research Center for Mental Disorders & Beijing Key Laboratory of Mental Disorders, Beijing Anding Hospital & the Advanced Innovation Center for Human Brain Protection, Capital Medical University, Beijing, China.; 5 Department of Neurosurgery, The Affiliated Hospital of Southwest Medical University; Neurosurgery Clinical Medical Research Center of Sichuan Province, Academician (Expert) Workstation of Sichuan Province; Sichuan, China.; 6 School of Nursing, Hong Kong Polytechnic University, Hong Kong SAR, China.; 7 Department of Psychiatry, The Melbourne Clinic and St Vincent's Hospital, University of Melbourne, Richmond, Victoria, Australia.

**Keywords:** COVID-19, psychiatric disorders

## Abstract

Recently, more than 300 Chinese patients with psychiatric disorders were diagnosed with the 2019 novel coronavirus disease (COVID-19). Possible reasons quoted in the report were the lack of caution regarding the COVID-19 outbreak in January and insufficient supplies of protective gear. We outlined major challenges for patients with psychiatric disorders and mental health professionals during the COVID-19 outbreak, and also discussed how to manage these challenges through further mental health service reform in China.

## Introduction

On 8 February 2020 an alarming report in the China News Weekly emerged that 1 at least 50 inpatients with psychiatric disorders and 30 mental health professionals in a major psychiatric hospital in Wuhan, Hubei province, China were diagnosed with the 2019 novel coronavirus disease (COVID-19). Possible reasons quoted in the report were the lack of caution regarding the COVID-19 outbreak in January and insufficient supplies of protective gear. On 18 February, 2020, the National Health Commission of China reported that 323 patients with severe psychiatric disorders were diagnosed with the COVID-19 2. To limit the transmission of the COVID-19 and provide acute treatment for severely ill patients, central and regional authorities have undertaken several effective measures, such as setting up emergency infectious hospitals and quarantine facilities, and isolating suspected and diagnosed patients and their close contacts 3. However, the current COVID-19 outbreak is presenting unique challenges and as a result, has many implications for psychiatric hospitals treating patients with major psychiatric disorders in China.

Due to the mandatory quarantine procedures in China, patients with suspected or diagnosed with COVID-19, close contacts and even frontline health professionals are at high risk of developing mental health problems 4. In response, the National Health Commission of China 5 has established crisis psychological intervention teams across many psychiatric hospitals in Hubei province and other parts of China 6. This inevitably places increased pressure on the already inadequate mental health resources in China. For instance, the proportion of psychiatrists and doctors in psychiatric hospitals was 2.15 per 100,000 in 2016 7, which is considerably lower than that in most developed countries 8. The mental health emergency response and deployment of expert teams from psychiatric hospitals during the COVID-19 outbreak may further deplete mental health services across psychiatric hospitals. In China, the public psychiatric hospitals are either run by the Ministry of Health, or by the public security and civil affairs systems. Apart from regular psychiatric services, psychiatric hospitals in the public security and civil affairs systems provide services in the areas of forensic psychiatry, substance dependence and psychiatric rehabilitation. Compared to those run by the Ministry of Health, these hospitals are usually located in suburban areas with poorer protective equipment and training for infectious diseases, which is associated with higher risk of the COVID-19 transmission. Some measures should be adopted to resolve these challenges. For example, outpatient visits need to be reduced in psychiatric hospitals, admission criteria should be tightened, and the length of hospitalization should be shortened 9.

Psychiatric inpatients in China may be more susceptible to severe viral outbreaks compared to patients in other health facilities. Patients in psychiatric hospitals are often confined to crowded living conditions in hospitals where they share common dining and bathroom spaces. Unlike general hospital patients who are usually nursed in hospital beds, psychiatric inpatients commonly participate in group activities which increase patient to patient contact. Due to their disordered mental state, poor self-control and self-care, and inadequate insight, they may be incapable of practicing infection control measures to protect themselves. Further, owing to the unhealthy lifestyle associated with mental illness and side effects of psychotropic medications, the suboptimal health status of hospitalized patients with major psychiatric disorders may render them more vulnerable to the COVID-19 pneumonia and its complications.

In China, most psychiatrists do not receive adequate training in the prevention and treatment of infectious diseases. Furthermore, in the past decade, internship period in internal medicine has been reduced for medical students whose major is psychiatry in many medical schools in order to extend their training in mental health. All these factors could limit their clinical competence to control the potential transmission of COVID-19 in psychiatric hospitals. In addition, the lack of community-based mental health services has led to an over-reliance on psychiatric services provided by large psychiatric hospitals 10, 11. For the vast majority of patients with serious mental illness, they can only access appropriate mental health care in hospital settings, which are mostly located in city areas. Therefore, they are more prone to exposure to infectious diseases such as the COVID-19, unless the psychiatric hospitals themselves implement stringent measures of infection control similar to general hospitals. Clearly, preventive measures such as the provision of adequate medical supplies and protective equipment, public education on the risks of COVID-19 for hospital staff and patients, and restricting family visits to hospitals, are essential to reduce the likelihood of disease transmission in major psychiatric hospitals. Moreover, all people including patients' families and health care workers need to be advised to wash hands regularly and maintain good personal hygiene. Measurement of body temperature should be performed at least once a day to closely monitor any suspected infectious symptoms. Activities involving group interaction in communal areas should be avoided. Moreover, in order to minimize the potential risk of nosocomial infection, isolation units should be established and hospital access should be restricted for inpatients 9.

The consequences of mass quarantines to contain the spread of the viral epidemic have highlighted the challenges of delivering psychiatric care to chronic patients with serious mental illness living in the community. According to the regulations of Chinese health insurance, clinically stable patients with major psychiatric disorders are usually required to visit hospital outpatient clinics, often far from their homes, to obtain their monthly maintenance medications. The suspension of public transportation in Wuhan and many other cities in China have created significant barriers for patients to access treatments and are likely to widen the treatment gap for serious mental disorders, at least in the short to medium term. To address the over-dependence on psychiatric hospitals, appropriate reform of the health insurance and development of community-based mental health services could increase the availability of psychotropic medications at community health clinics, and reduce patients' need to travel, thereby decreasing pressure on psychiatric hospital outpatient clinics. Furthermore, community psychiatric outreach services by psychiatrists and nurses should be developed for clinically stable patients. To avoid the need to attend hospitals, mental health professionals could use special designated vehicles to visit patients at home to assess their symptoms, and provide necessary medications. In addition, it is important to establish an infection disease alarm and control system in psychiatric hospitals. The health authorities should hire specialists to monitor, implement and adopt the preventive and control measures for infectious diseases, and establish infectious units in psychiatric hospital settings 12.

One of the largest community mental health projects globally is the mental health care model in China entitled the “Management and treatment program for severe mental illness”. In order to establish community-based mental health services nationwide, this project integrated the resources in psychiatric hospitals and existing community psychiatric services and trained mental health professionals in the development of individual service plans 13. In the last decade, regular maintenance treatments have been provided for millions of community-dwelling patients with severe psychiatric disorders in this project, particularly for those with relatively high risk of violent behaviours. Regular outreach psychiatric treatment, rehabilitation and prevention services are provided under this national program 11. Of note, this program was initially developed as part of the national effort to rebuild the community health system following the 2003 severe acute respiratory syndrome (SARS) outbreak in China. Although there has been considerable progress and investment in the mental health system following this, there remain many aspects of the community mental health service system that need strengthening as can be observed in the service gaps accentuated by the recent health crisis in China.

**Figure 1:**
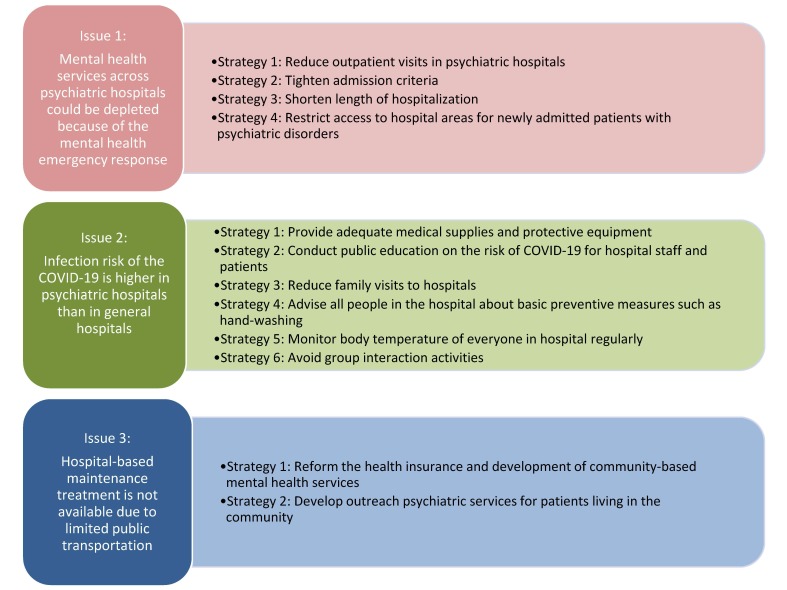
Strategies to improve mental health services during the outbreak of the COVID-19 in China.

Finally, managing patients with severe psychiatric disorders who have suspected or confirmed COVID-19 has created a major logistical challenge. Although a 30-bed ward in an infectious disease hospital in Wuhan was established for psychiatric patients on 3 February 2020, the rapidly escalating number of cases has led to a severe shortage of hospital beds. As an alternative, isolation wards have been established in psychiatric hospitals for psychiatric patients with suspected and confirmed COVID-19, but this option could increase the risk of hospital-acquired infection especially if there is inadequate capacity for infection control in these hospitals 1. Another option is to establish specific quarantine facilities for clinically stable psychiatric patients with mild-moderate symptoms of COVID-19 infection. Gymnasiums, exhibition centers and sports centers have been converted into '*Fang Cang*' (temporary quarantine hospital facilities) for infected patients with mild symptoms 14 (Figure 1).

In conclusion, the COVID-19 outbreak has raised numerous challenges for psychiatric hospitals in China to safely manage patients' major psychiatric disorders in addition to preventing and treating COVID-19. In addressing these challenges, future community mental health system reform is necessary to re-balance the system by re-distributing resources from hospital-centric services to community-based and primary care services. Lessons and experiences from previous bio-disasters such as SARS that have led to the strengthening of the public mental health system should be considered.
